# Large-scale surgical workflow segmentation for laparoscopic sacrocolpopexy

**DOI:** 10.1007/s11548-021-02544-5

**Published:** 2022-01-20

**Authors:** Yitong Zhang, Sophia Bano, Ann-Sophie Page, Jan Deprest, Danail Stoyanov, Francisco Vasconcelos

**Affiliations:** 1 Wellcome/EPSRC Centre for Interventional and Surgical Sciences (WEISS) and Department of Computer Science, University College London, London, UK; 2 Department of Development and Regeneration, University Hospital Leuven, Leuven, Belgium

**Keywords:** Surgical workflow segmentation, Machine learning, Laparoscopic sacrocolpopexy, Long short-term memory networks, Transformer networks

## Abstract

**Purpose:**

Laparoscopic sacrocolpopexy is the gold standard procedure for the management of vaginal vault prolapse. Studying surgical skills and different approaches to this procedure requires an analysis at the level of each of its individual phases, thus motivating investigation of automated surgical workflow for expediting this research. Phase durations in this procedure are significantly larger and more variable than commonly available benchmarks such as Cholec80, and we assess these differences.

**Methodology:**

We introduce sequence-to-sequence (seq2seq) models for coarse-level phase segmentation in order to deal with highly variable phase durations in Sacrocolpopexy. Multiple architectures (LSTM and transformer), configurations (time-shifted, time-synchronous), and training strategies are tested with this novel framework to explore its flexibility.

**Results:**

We perform 7-fold cross-validation on a dataset with 14 complete videos of sacrocolpopexy. We perform both a frame-based (accuracy, F1-score) and an event-based (Ward metric) evaluation of our algorithms and show that different architectures present a trade-off between higher number of accurate frames (LSTM, Mode average) or more consistent ordering of phase transitions (Transformer). We compare the implementations on the widely used Cholec80 dataset and verify that relative performances are different to those in Sacrocolpopexy.

**Conclusions:**

We show that workflow segmentation of Sacrocolpopexy videos has specific challenges that are different to the widely used benchmark Cholec80 and require dedicated approaches to deal with the significantly larger phase durations. We demonstrate the feasibility of seq2seq models in Sacrocolpopexy, a broad framework that can be further explored with new configurations. We show that an event-based evaluation metric is useful to evaluate workflow segmentation algorithms and provides complementary insight to the more commonly used metrics such as accuracy or F1-score.

**Supplementary Information:**

The online version supplementary material available at 10.1007/s11548-021-02544-5.

## Introduction

Half of the women above 50 years of age suffer from pelvic organ prolapse, with a lifetime prevalence of $30\pm 50$% [24], and in particular, vaginal vault prolapse is a frequent occurrence after hysterectomy [10]. This condition occurs when the vaginal vault sags down from its position, causing discomfort and urinary problems to the patient. Sacrocolpopexy has been regarded as the gold standard procedure for its treatment [21], and while it can be performed with either open or minimally invasive laparoscopic approach, the latter is associated with lower morbidity rates and recovery periods [12].

**Fig. 1:**
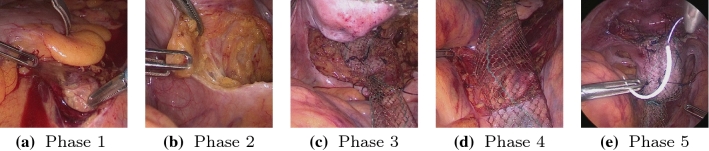
Surgical phases of laparoscopic sacrocolpopexy with average duration in seconds: 1) promontory preparation ($633\pm 365$); 2) dissection of vault and gutter ($3097\pm 1212$); 3) mesh fixation to vault ($3888\pm 879$); 4) mesh fixation to promontory ($211\pm 157$); 5) peritonealisation ($1073\pm 548$)

Laparoscopic sacrocolpopexy aims at fixating the vaginal vault using a mesh implant that is permanently sutured or stapled to the sacral promontory. The workflow of this procedure can be divided into 5 phases (Fig. 1): 1) dissection of the promontory; 2) dissection of vaginal vault and gutter; 3) implant fixation to the vault; 4) implant fixation to the promontory; 5) peritonealisation. This procedure requires a significant amount of training [10]. Claerhout et al. [6] show that the operation time declines rapidly after the first 30 procedures and at a slower rate until 90 procedures are attained. The learning rate for each particular phase is also different, and the dissection of the promontory is regarded as the most difficult to master [7]. Additionally, the different approaches for attaching the mesh implant mean that some surgical phases can be modified and need to be analysed individually to investigate surgical skills. As different approaches to sacrocolpopexy are introduced, larger studies become necessary to understand how they affect surgical learning rates at the level of each phase. However, going through large volumes of surgical video to annotate the duration of each phase is significantly time-consuming and requires extensive dedicated time from clinically trained staff. Automating this task would greatly assist in the investigation of surgical skills and the impact of procedural changes in sacrocolpopexy, and this can be accomplished with surgical workflow segmentation.

**Fig. 2:**
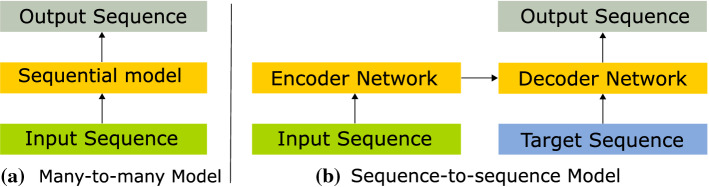
Network architectures for coarse-level sequential models. The main differences from the sequence-to-sequence to the many-to-many model are: 1) the presence of an encoder-decoder structure, allowing input/output sequences to have different sizes; 2) In addition to a sequence of feature vectors (input sequence), the input to this model also includes a sequence of label classifications (target sequence). The colour legend can be referred to Fig. 2

In most image-guided surgeries, the main source of information for workflow segmentation is video. However, this can be complemented with additional data, such as instrument trajectories from robot joint kinematics [1, 20] or activity signals from surgical instruments [29]. In the case of Sacrocolpopexy, the only available source of information is the laparoscopic video, and therefore, we will review approaches under this constraint. A few works rely on tool detection as a prior to surgical phase recognition. While this has shown to be a successful strategy in cholecystectomy [4, 17, 26] and cataracts [33], it relies on the availability of groundtruth tool labels and therefore requires either time-consuming tool annotation or additional measurements in the operating room. In many circumstances, such as with our Sacrocolpopexy data, we are limited to temporal phase annotations alone.

The state-of-the-art in this domain is based on supervised deep learning. Given the temporal nature of this problem, it is typically approached using a fine-level convolution network that estimates surgical phases from a single frame (CNN) or a short sequence of frames (3D-CNN), followed by a coarse-level temporal model that refines phase segmentation using constraints at a larger timescale (e.g. Long Short-Term Memory (LSTM) networks [4, 17], HMM [26], Temporal Convolutional Networks [8]). Defining the scope of fine and coarse features in Sacrocolpopexy is a challenge due to the extreme variations in procedural time, ranging from around 2 to 5 hours in total. Similarly, phase duration can vary from 1 minute (mesh fixation to promontory) to 4 hours (dissection of vault and gutter). This highly contrasts with popular public datasets in cholecystectomy and cataracts that have more stable workflow patterns [26, 33]. Therefore, we are interested in a scalable solution that can capture temporal details with highly varying coarse intervals.

In this paper, we introduce sequence-to-sequence (seq2seq) learning as a flexible framework for modelling coarse temporal workflow features. Seq2seq was popularised in natural language processing for text-to-text conversions such as language translation [13, 15], but has also been recently used to model temporal features in video analysis [30, 31]. Unlike previous approaches to coarse workflow modelling with LSTM, seq2seq allows for input-output sequences with independent and arbitrary dimensions that could, e.g., be utilised in forecasting future workflow events [5, 32]. To the best of our knowledge, this formulation has not been applied before to workflow segmentation, and therefore, we perform an ablation study on multiple seq2seq configurations and learning strategies to explore its potential in this domain. Additionally, we introduce event-based accuracy metrics [28] to surgical workflow segmentation. While algorithms in this domain are typically evaluated in terms of aggregate classification outcomes (precision, recall, etc), this is not completely informative if we aim at estimating timestamps for certain events during the procedure, such as a phase transition, from noisy segmentation results. Other considerations related to the number of erroneous classification transitions need to be analysed.

Our main contributions can be summarised as following:

- Introducing workflow segmentation in the context of laparoscopic sacrocolpopexy, with its significant challenges in terms of large and highly varying phase duration. These differences are also highlighted in comparison with the widely used benchmark Cholec80.
- A general seq2seq formulation of the surgical workflow segmentation problem and several implementations with different configurations (time-synchronous and time-shifted), architectures (LSTM [14] and transformer [27]) and learning strategies. The time-shifted configuration has the advantage of not requiring a fine-level initialisation beyond the first few frames of a video.
- An event-based evaluation methodology for surgical workflow that complements standard classification metrics to inform on potential workflow applications such as automated time-stamping of events.
- The code in this work can be downloaded at: https://github.com/yitongzh/Sacrocolpopexy-workflow-analysis

## Sequence-to-sequence (seq2seq) models

Surgical workflow segmentation is a sequential multi-label classification problem with inherent temporal constraints. Considering the most recent state-of-the-art deep learning approaches, these temporal constraints can be modelled at a fine-level with 3D convolutional neural networks (3D CNN’s), and at a coarse level with a recurrent model, such as LSTM. In this section, we assume that a fine-level model estimates a sequence of feature vectors (input sequence) and an initial workflow segmentation from them (target sequence). We will now explore different ways of processing these sequences at a coarse level to produce an output sequence that represents our final workflow segmentation.

We refer to conventional recurrent models in this domain as many-to-many models, since both their input and output are sequences with the same dimension, with a one-to-one correspondence. In contrast, a seq2seq model can have input and output sequences with different sizes that are linked by an encoder-decoder architecture. Additionally, a seq2seq model uses the fine-level predictions (target sequence) to guide feature selection at the the decoder level. These differences are summarised in Fig. 2. Recent works have also used related strategies for feature selection though attention mechanisms in the context of cholecystectomy workflow segmentation [9, 11].

**Fig. 3:**
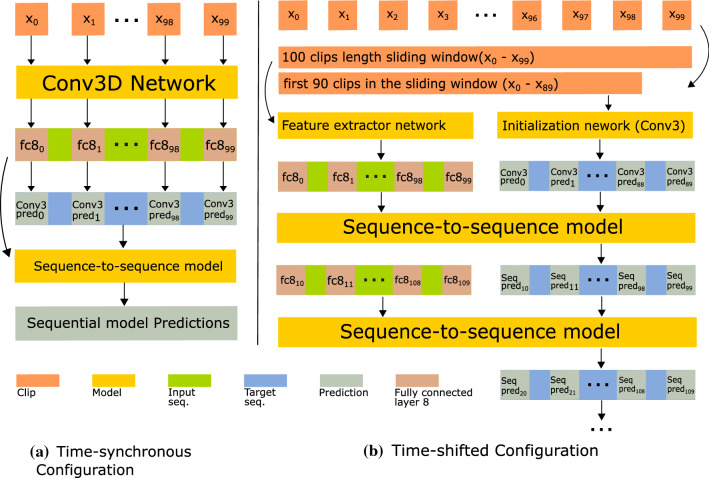
Seq2seq Network Architecture with a sequential input of 100 clips. The length of the target and output sequence depends on the configuration of the network: **a** in the time-synchronous configuration, the target, input and output sequences correspond to the same time interval of 100 clips; **b** in the time-shifted configuration, the target and output sequences have a length of 90 time steps with a shift of 10 between them. Together they span a length of 100 clips, which corresponds to the size of the input sequence that is obtained from the Conv3D feature extractor. To obtain segmentations for consecutive sequences in a video, the seq2seq predictions become the target sequence of the next prediction iteration

We investigate two potential configurations of the seq2seq model: time-synchronous and time-shifted (Fig. 3). With the time-synchronous configuration, the sequence of input labels (target sequence), input feature vectors (input sequence), and final classifications (output sequence) all correspond to the same time interval. This usage works as a global refinement of a sequence that was previously estimated with a fine-level method (e. g. CNN, 3D-CNN). This is most similar to the conventional many-to-many recurrent models, but it explicitly requires a fine-level model to provide both a sequence of features and its segmentation results (many-to-many only requires features). On the other hand, the time-shifted configuration does not require fine-level segmentation results beyond the first sequence in the video. This is achieved by having the target sequence shifted (to the past) relative to input/output sequences (in the present) by a fixed number of time-steps. Therefore, if a workflow segmentation is obtained by sliding a sequence along an entire video, the target sequence can be obtained exclusively at coarse-level using past seq2seq predictions, instead of relying on fine-level predictions like the time-synchronous configuration. We also note that this is different from predicting future labels from present information, given that the input and output sequences still correspond to the same time interval.

## Proposed network architectures

Our proposed network (Fig. 3) has two main components: a 3D convolutional neural network (Conv3D) for fine level phase classification and a seq2seq model for coarse level refinement. Conv3D takes clips of 16 RGB consecutive images with 112$\times$112 pixel resolution. The 3D convolution layers follows the EndoNet architecture [4] which is based on Alexnet [19]. A final fully connected layer is added to output 5 classifications (phases in sacrocolpopexy). Both the final classification and the 1200 dimensional feature vector from the previous fc8 layer is fed into the seq2seq model.

The seq2seq model analyses a larger video segment, consisting of 100 Conv3D clips. The base unit of seq2seq sequences are clips, not frames. Therefore, at a coarse level, we refer to the label of an entire clip as the most frequent label in its 16 frames. During network training, the target sequence can be defined differently, e.g. as the groundtruth labels. We implement seq2seq with two base architectures, LSTM [14] and transformer [27]. Note that LSTM has been already used for surgical phase segmentation [16, 22, 33], but only as a conventional many-to-many sequential model. In this paper, we refer to LSTM adapted to the seq2seq structure.

We additionally consider two configurations: time-synchronous and time-shifted. The *time-synchronous* configuration (Fig. 3a) takes as the target sequence the 100 labels corresponding to the same clips as the fc8 feature vectors. Hence, these networks are named as LSTM100(L100) and Transformer100(T100) for simplicity. The *time-shifted* configuration (Fig. 3b) takes as the target sequence only the first 90 labels corresponding to the 100 fc8 feature vectors to predict the last 90 labels of that sequence. Hence, there are 10 labels in the prediction that act as ’future’ labels relative to the target sequence with only 80 overlapping labels. By having this shift between target sequence and prediction, the detection can have the first 90 target sequence to be initialized by the Conv3D network and the prediction of all following labels in the video relies completely on the seq2seq model by treating the previous predictions as the current target sequence recursively. These types of networks are named as LSTM90(L90) and Transformer90(T90) for simplicity.

### Network parameters

The detailed network parameters of Conv3D are presented in the supplementary document. The fc8 layer that is extracted for sequential model input has a dimension of 1200. This dimension is used as the hidden dimensions of the LSTM model for convenience, and each separate LSTM model (many-to-many, LSTM encoder and LSTM decoder) has 3 hidden layers.

The transformer setup is analogue to the originally proposed default settings [27] with 6 layers of the 8 head encoder-decoder pairs. The input dimensions are adjusted to fit our input sequence with a sequence length of 100 and the $d_{\mathrm{model}}$ of 1200. The inner layer dimension for the feed forward network is reduced to 1000 to decrease the model size. And the sine and cosine functions of different frequencies are used for positional encoding, as in [27].

### Network training strategies

Conv3D and Seq2seq are trained separately. The Conv3D model was fine-tuned based on the parameters that have been pre-trained on the Cholec80 dataset, as Cholec80 and our dataset have roughly similar tool usages and the tissue shares some spatial features. For seq2seq, we defined different training strategies in terms of sampling policy and usage of the target sequence. All strategies are independently modified from a baseline so that an ablation study can evaluate them independently. These training strategies are defined as following:

- **Standard method (baseline)**: During training, the input to the target sequence is the groundtruth labels. When deployed, the network uses Conv3D (time-synchronous) or past Seq2Seq (time-shifted) predictions instead. This is the standard approach for training seq2seq models in previous works [25]. The entire video is sampled for training in sequence. For balancing the training data, a fixed number of sliding windows (200) is sampled from each video, with their interval changing depending on total video time. During training, the sliding windows with the same indices will be extracted from each video and assembled into a batch. Hence, each batch contains the samples that are at the same relative positions in all videos.
- **Target Sequence with injected noise (noised)**: We inject noise into the groundtruth target sequence to simulate prior classification errors during training and enabling seq2seq to learn a filtering action. Noise is injected by randomly replacing 40% of correct labels.
- **Target Sequence with Predicted Labels (pred)**: Similarly to the previous strategy, we introduce classification errors by using Conv3D predicted labels as the target sequence. This method may preserve some internal structures between the predicted labels.

### Loss function

Cross entropy loss is utilized in training the network. The general form of the loss function for the Conv3D network is:

1 $$\begin{aligned} L_{\mathrm{Conv}}(y,x) = \frac{1}{d} \sum _{j=1}^d \sum _{i=1}^n w_i y_{i,j} log(x_{i,j}), \end{aligned}$$

where *x* is the softmax output from the network and *y* is the one-hot label for that particular clip. There are *n* classes of labels that represent the phases, and each label has a corresponding weight $w_i$ in evaluation. Multiple samples are trained together with a batch size *d*, and the average loss for all samples is considered as the general loss for that batch.

The loss function for the sequential model is similar, but with an extra time dimension *t* for the sequence length:

2 $$\begin{aligned} L_{\mathrm{sequential}}(y,x) = \frac{1}{td} \sum _{k=1}^t \sum _{j=1}^d \sum _{i=1}^n w_i y_{i,j,k} log(x_{i,j,k}). \end{aligned}$$

## Experimental setup

### Dataset

The dataset contains 14 videos of laparoscopic sacrocolpopexy surgery performed by the same group of surgeons. The videos are acquired at 24 fps resolution with a display resolution of $1920\times 1080$ pixels. Each video captured a complete procedure, where the average duration was 3 hours 13 minutes, with the shortest video of 1 hours and 47 minutes and the longest video of 4 hours 56 minutes. Each video was annotated by an expert Gynaecologist to indicate the start, the end and any pausing and resuming of each phase as timestamps.

Besides the five phases of the procedure, some frames are labelled as transition and non-phase. The transition phase is defined as the moment when the previous phase finishes, but the next phase has not begun yet. These are relatively few and short but could potentially aid in classifying previous and subsequent phases within a temporal window. In the training loss function, we attribute the transition phase a weight that is 10 times smaller than the other phases to avoid its overestimation. The non-phase refers to the moment before the first phase and after the last phase, and we exclude it from the analysis. The five phases shown in Fig. 1 are the major phases that are needed for the surgical workflow analysis and skills assessment.

### Post-processing

Both the time-synchronous configuration and the time-shifted configurations have fixed-length input and output sequences. The length is designed to be short enough for extracting sufficient amount of sliding windows from the videos. Hence, it is necessary for composing the output sequence together for a final predicted sequence. For the time-shifted configuration, there are overlaps existing between the sliding windows. A single time step in the video can have multiple predictions throughout the sliding windows. A mode filter is applied to each time step for the final prediction. For the time-synchronous configuration, the sliding windows can be assembled in sequence as there are no overlaps between them.

### Comparison with the state-of-the-art

With the sacrocolpopexy dataset, we compare our seq2seq results against raw predictions from [4] (C3D), a filtered version with mode averaging, and the many-to-many models LSTM and TCN. C3D+LSTM can take sequences of arbitrary length, and thus, it is normal to perform predictions based on all past frames. However, our seq2seq models require a fixed sized sequence and perform predictions using a sliding window. To understand how this affects the performance, we test C3D+LSTM with both all past frames and with a sliding window. All above methods are also tested on the Cholec80 public dataset [26] to which we add for completeness the state-of-the-art results as reported in [4, 8, 17]. The major difference between our dataset and Cholec80 is the overall duration of each phase, which can be significantly larger in Sacrocolpopexy. Notably, this significantly changes the relative performance between different algorithms, as we show in Results and discussion section.

### Training details

The captured videos are downsampled to 2.4 fps, centre cropped, and resized into a square of resolution $300 \times 300$ pixels. Then, 16 consecutive frames are assembled into a clip as the basic unit of input for the Conv3D network. The most common label (mode value) for all the frames in a clip is assigned as the label for that clip. The sequential model takes a continuous sequence of 100 clips (1600 frames) as input, where the clips are processed by the Conv3D network first and its last fully connected layer of 1200 neurons for those 100 clips are assembled into a tensor as one training sample.

Data augmentation is applied to each clip along with sampling [3] by performing horizontal and/or vertical flip, rotation in the range of 0 to 360 degrees, crop with a minimum factor of $\frac{1}{9}$ of the original image and then resizing, blur with a Gaussian filter of $5 \times 5$ kernel with 1.5 standard deviation and luminance variation in the range of 0.6 to 1.4. These augmentations are selected randomly with a uniform distribution within the indicated ranges. The same augmentation is applied to all the frames in a single clip for consistency. Finally, all frames are resized to $112 \times 112$ pixels to match the input requirement of the network. The proposed network is implemented in PyTorch using a single Tesla V100-DGXS-32GB GPU of an NVIDIA DGX station.

The training is performed using 7-fold cross-validation. The 14 videos that constitute our dataset are divided into seven pairs where five of them are used for training, 1 pair is used for validation and 1 pair is used for testing. Adam [18] optimiser with a learning rate ($l_r$) of $1e^{-5}$ and a decay set to $0.93 \times l_r$ for every fifth epoch is used for the Conv3D network training. Each epoch contains 600 samples of batch size 10 with each phase sampled to a same number. The average accuracy without the transition phase and non-phase is calculated on the validation set 4 times per epoch, and network parameters with the best accuracy in history are saved as the final parameters. The output (fc8 and prediction) of the trained Conv3D is then used as input for sequential models.

### Evaluation metrics

Two evaluation are performed. The first one is the frame-based macro-averaged precision and recall that gives the overall performance. The transition phase is excluded for its unimportance in the calculation of averaging over phases. F1-score is also calculated through the macro-averaged precision and recall. As the evaluation metrics used in most of other works, the accuracy here is calculated based on the whole videos rather than a per phase macro-average. The second evaluation is done with the Ward metric [28] which provides in-depth analysis of the error by further breaking it down into sub-categories. The Ward metric is shown to be effective for evaluating sequential predictions, e.g. video or sensor-based activity recognition [2, 28]. There are two types of Ward metrics: frame-based and event-based. We focus on the event-based in this paper, which evaluates the event as a continuous positive label with a start time and a stop time. It defines 5 sub-categories of error by comparing the predicted sequence to the ground truth sequence as: deletion(D), insertion(I$'$), merge(M, M$'$), fragmentation(F, F$'$), and Fragmented and Merged(FM, FM$'$), where the prime symbol indicates the segment in the predicted sequence. The Ward metric counts each type of error individually and summarizes them in an event analysis diagram (EAD). In our research, as a multi-class classification problem, we implemented the ward metric phase-by-phase and added them up to obtain the final evaluation of a single sequence. We also define an event ratio calculated by the event number of the groundtruth over the event number of the prediction sequence. An event ratio close to 0 suggests the worst performance, while a value close to 1 suggests that the event number in groundtruth and predictions are the same.

## Results and discussion

**Table 1:** Ablative phase recognition results(%) over different proposed architectures on Sacrocolpolpexy dataset the best among each configuration are shown as italic for 100 series and bolditalic for 90 series

Architectures			Precision (Macro)	Recall (Macro)	F1-Score	Accuracy (Micro)
LSTM(L)	100	Baseline	61.6 ± 6.7	74.8 ± 9.7	0.68	70.7 ± 9.0
		Pred	72.8 ± 12.8	69.6 ± 17.6	0.71	80.4 ± 13.0
		Noised	74.6 ± 11.8	*78.8 ± 11.5*	*0.77*	*82.8 ± 9.8*
	90	Baseline	53.7 ± 24.1	54.4 ± 17.5	0.54	67.2 ± 22.3
		Pred	57.7 ± 16.0	59.0 ± 15.1	0.58	75.5 ± 20.2
		Noised	53.5 ± 16.3	58.8 ± 11.7	0.56	76.5 ± 16.0
Transformer(T)	100	Baseline	64.6 ± 13.7	63.2 ± 14.7	0.64	73.1 ± 13.4
		Pred	*75.4 ± 14.3*	69.4 ± 14.2	0.72	80.6 ± 16.1
		Noised	72.9 ± 14.2	68.6 ± 15.7	0.71	82.7 ± 13.5
	90	Baseline	***76.4 ± 12.6***	***71.7 ± 15.5***	***0.74***	81.1 ± 15.5
		Pred	71.7 ± 14.2	65.1 ± 13.1	0.68	80.4 ± 14.1
		Noised	74.9 ± 13.6	71.2 ± 15.5	0.73	***81.9 ± 14.1***

Bold values indicate the best performance

**Fig. 4:**
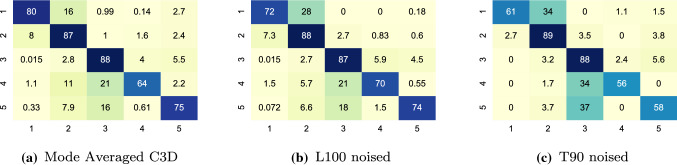
Sacrocolpopexy per phase results: averaged confusion matrices(%) over all cross-validation folds normalized by the sample number of each phase with the two best methods in sequential models. (Note: transition phase is eliminated from the graph)

**Fig. 5:**
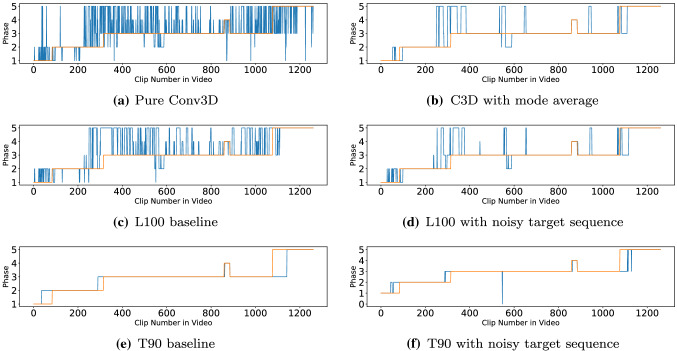
Phase diagrams for six different configurations, showing output sequences from the best Sacrocolpopexy fold. Groundtruth (blue) and predicted (orange) labels are shown

Table 1 shows an ablation study of our different seq2seq implementations, and Fig. 5 shows its results on a particular video sequence. The noised training strategy overall performed best for both time-synchronous and time-shifted configurations, with, respectively, LSTM100 (L100) and Transformer90 (T90) being the best performing in terms of accuracy. The baseline strategy using groundtruth labels for the target sequence is generally the worst, with a single exception (T90). In this case, the network suffers from the exposure bias [23] as there is a strong dependency between the groundtruth and the predictions.

Figure 4b, c provide the confusion matrices of the selected methods. Most of the misclassifications for this type of surgery happens between the two consecutive phases as phase 1-2, phase 3-4 and phase 3-5. The mesh is introduced in phase 3 which separates the following phases from the first 2 phases. The same tools are also used in phase 3, 4 and 5 but applied to different positions with phase 3 (promontory) and 4 (vault). Phase 5 can be started from either phase 3 or 4 but in most cases is phase 3, hence it has more misclassifications with phase 3 rather than phase 4.

**Table 2:** Comparison of the phase recognition results(%) with other methods on the Sacrocolpopexy and Cholec80 datasets

	Sacrocolpopexy (average of 1389 clips per video)				Cholec80 (average of 360 clips per video)		
Method	Pre. (Macro)	Rec. (Macro)	F1-Score	Acc. (Micro)	Pre. (Macro)	Rec. (Macro)	Acc. (Micro)
C3D+LSTM+Tool(Endo3D)*[4]					81.3	**87.7**	91.2
ResNet-50+LSTM+PKI (SV-RCNet)*[17]					**90.6 ± 8.1**	86.2 ± 15.3	**92.4 ± 5.2**
ResNet-50+LSTM *[17]					80.7 ± 7.0	83.5 ± 7.5	85.3 ± 7.3
ResNet-50+TCN(TeCNO Stage I)*[8]					**82.44 ± 0.46**	**84.71 ± 0.71**	**88.35 ± 0.3**
C3D	58.5 ± 6.8	68.6 ± 10.1	0.63	69.2 ± 8.8	67.5 ± 8.1	74.7 ± 7.4	71.0 ± 8.5
C3D + Mode average	*78.1 ± 9.5*	*79.7 ± 12.6*	*0.79*	*82.8 ± 9.5*	73.9 ± 10.6	81.2 ± 9.9	79.5 ± 8.1
C3D+TCN	76.6 ± 12.6	74.3 ± 15.3	0.72	82.6 ± 12.4	81.3 ± 5.9	***82.0 ± 8.4***	83.8 ± 7.8
C3D+LSTM	71.6 ± 22.6	64.8 ± 19	0.68	77.1 ± 18.8	***80.1 ± 10.0***	***82.0 ± 8.3***	***85.9 ± 7.9***
C3D+LSTM+Sliding Window	71.2 ± 17.5	65.8 ± 15.7	0.68	79.2 ± 14.7			
C3D+T90 noised(Proposed)	74.9 ± 13.6	71.2 ± 15.5	0.73	81.9 ± 14.1	43.7 ± 18.7	48.1 ± 16.0	71.1 ± 13.9
C3D+L100 noised(Proposed)	74.6 ± 11.8	78.8 ± 11.5	0.77	*82.8 ± 9.8*	64.9 ± 9.6	73.5 ± 10.6	81.1 ± 5.3

Bold and Bold italic values indicate the best performance

Asterisk (*) denotes cholec80 results were directly extracted from respective publications, while the others are our own implementations. This table is grouped by (row 1–2) methods that use models specific to cholecystectomy (tools or priors), as reported in previous literature; (row 3–4) models with ResNet-50 backbone, as reported in previous literature; (row 5–11) models with a C3D backbone, as proposed in this paper

Our best performing seq2seq time-synchronous and time-shifted models (T90, L100 noised) are also compared with previously proposed approaches on our Sacrocolpopexy dataset (Table 2). First, we can observe that performing predictions on a sliding window does not affect the general performance of C3D+LSTM, slightly increasing its accuracy. This suggests that the loss of input information from using a fixed sliding window is not negatively affecting performance, and therefore, this should not be a limiting factor in our seq2seq architectures that always operate on a sliding window. Both seq2seq models (T100,L90) outperform the many-to-many approach (C3D+LSTM). Surprisingly, the best performance in terms of F1-score is the simple mode average on C3D results, which narrowly beats the seq2seq L100 noised. However, an analysis purely based on F1-scores disregards how accurately are we capturing a time ordered sequence of events. To further interpret these results, we also perform an event-based evaluation.

**Table 3:** Ward Metric results summed over all Sacrocolpopexy cross-validation folds

Method	F	C	F’	event ratio
C3D	79	4	2299	0.015
C3D+Mode Average	49	33	218	0.172
C3D+TCN	66	15	578	0.072
C3D+LSTM(no-tool)	30	41	123	0.287
C3D+LSTM+sliding window	39	35	150	0.237
L100 noised(Proposed)	63	19	415	0.098
T90 noised(Proposed)	**28**	**42**	**98**	**0.342**
LSTM avg.	40	24	347	0.217
Trans avg.	36	34	188	0.215
100 series avg.	54	22	427	0.123
90 series avg.	22	36	107	0.309

Bold values indicate the best performance in each category

F and F$'$ represents the fragmentation label where an event F in the groundtruth is fragmented into multiple F$'$ events in the predictions. C represents the correct labels for the events in predictions that are matched with the corresponding events in ground truth. This table is grouped by (row 1-7) the Ward metric of our tested methods and (row 8-11) the average Ward metric over each category of our proposed methods

Table 3 shows the sum of the Ward metric results over the 7 cross validation folds. The event ratio, number of correct (C) events and number of the fragmentation errors (F, F$'$) are presented in the table. A higher event ratio means that the temporal order of phase transitions is better preserved. Filtering very noisy predictions generally leads to better results in this evaluation due to eliminating a significant number of false phase transitions (e.g. comparing C3D with its mode average). Seq2seq models can further increase the event ratio in most cases. T90 noised has a slightly worse F1-score and accuracy than the mode average, but it has a significantly better Ward metric, specifically in terms of its event ratio and low fragmentation number. This effect can be visualised in the example results in Fig. 5, where even if overall accurate, mode average tends to have many incorrect transitions, while T90 performs all transitions in correct order but accumulates errors near the phase transitions. This may be a desirable outcome, since phase transitions are by definition more subject to annotation ambiguity than the middle of the phases. The trade-off between F1-score and event ratio can also be observed by comparing the overall performance of time-synchronous configurations (100 series) with the time-shifted configurations (90 series). The first one tends to perform better in terms of F1-score, while second better preserves number and order of transitions.

Table 2 also compares the performance of networks on the Cholec80 dataset. We focus our comparisons on the temporal models for a common backbone (C3D). We also display results based on ResNet-50 as reported in original publications for reference. C3D+LSTM achieves a close result to the original C3D+LSTM+Tool, where the slight decrease in performance is explained by not using tool signal information. The average number of clips per video in Cholec80 is 360 which is much smaller than in Sacrocolpopexy (1389). Furthermore, the relative proportions of each phase are also generally different. Taking these factors into consideration, it is worth noting that the relative performances between our implemented methods are almost reverted in Cholec80, with C3D+LSTM performing the best and mode average the second worst. This shows that the specific characteristics of a given surgery greatly affect algorithm performance. More specifically, we verify that our seq2seq models outperform LSTM and TCN on Sacrocoplopexy but this is not the case on Cholec80. When we compare the same temporal models (LSTM, TCN) either using C3D or ResNet, the results do not differ significantly, reinforcing again that the main differences are observed when applying the same methods to the different datasets. We should also highlight ResNet+LSTM+PKI, despite having the best results on cholec80, uses priors specific to cholecyestctomy surgery as a pre-processing step, and therefore cannot off-the-shelf be applied to our Sacrocolpopexy dataset.

## Conclusion

In this paper, we introduce seq2seq models as a novel coarse-level sequential model for surgical workflow segmentation. We validated the approach on a challenging dataset of Sacrocolpopexy surgery, where phase duration has a very high variability. We experimentally highlight the differences between this dataset and the widely studied benchmark Cholec80, showing that the same set of algorithms have different relative performances on each dataset. We observed that the for the statistical results on Sacrocolpopexy, unlike the results of Cholec80, the standard deviations are large and numerous results are not significantly different from each other. Especially for our proposed method (T90 noised) and the mode average result. Thus, we introduced an event-based analysis (Ward metric) to complement more standard accuracy metrics (F1-score,accuracy). The method evaluates the sequences on consistent temporal events. Our purposed method detected the highest number of correct events and has the highest event ratio, whereas the mode average method predicted fewer corrected events but with a much lower event ratio. The event ratio revealed the consistency of the detected events as more fragmentations and insertions will yield a lower event ratio. This observation suggests that despite some methods performed well in standard accuracy metrics, the quality of the output sequence is still limited. How each criterion should be weighted will invariably be application specific. Nonetheless, accurate time-stamping of phase transitions requires both standard and event metrics to perform well

There are several improvements that can be pursued in future research. The convolution neural network and the sequential model are trained separately, but could be fine-tuned in an end-to-end fashion. Many seq2seq configurations remain unexplored, for example input and output sequences with different sizes could produce even more coarse segmentations from dense input sequences, and could potentially further address scalability issues. Modelling surgery-specific priors can improve predictions on Cholec80 (SV-RCNet+PKI [17]) and similar strategies could be developed for Sacrocolpopexy. Temporal convolutional networks [8] have also shown promising results in surgical workflow segmentation and can be potentially considered within the seq2seq framework.

As for the currently available data, all video recordings were successful operations by the same surgeon, and thus, we expect that the challenges due to high variability in phase duration will only increase if more heterogeneous data are included. This further justifies exploring the flexibility of seq2seq models in this context.

## Supplementary Information

Below is the link to the electronic supplementary material.

Supplementary material 1 (pdf 108 KB)
