# Supplementary material for: Large-scale surgical workflow segmentation for laparoscopic sacrocolpopexy
Source: Int J Comput Assist Radiol Surg. 2022 Jan 20;17(3):467–77. doi: 10.1007/s11548-021-02544-5 (PMC8873061; doi:10.1007/s11548-021-02544-5)
Supplement: Supplementary file 1 — Supplementary material 1 (pdf 108 KB) [file 11548_2021_2544_MOESM1_ESM.pdf]

Noname manuscript No.
(will be inserted by the editor)

Large-scale surgical workflow segmentation for Laparoscopic Sacrocolpopexy

Yitong Zhang · Sophia Bano · Ann-Sophie Page · Jan Deprest · Danail Stoyanov · Francisco Vasconcelos

Received: date / Accepted: date

Y. Zhang, S. Bano, F. Vasconcelos, D. Stoyanov
Wellcome/EPSRC Centre for Interventional and Surgical Sciences(WEISS) and Department of Computer Science, University College London, London, UK
Ann-Sophie Page, Jan Deprest
Department of Development and Regeneration, University Hospital Leuven, Leuven, Belgium

Supplementary Document:

1 Conv3D Architecture

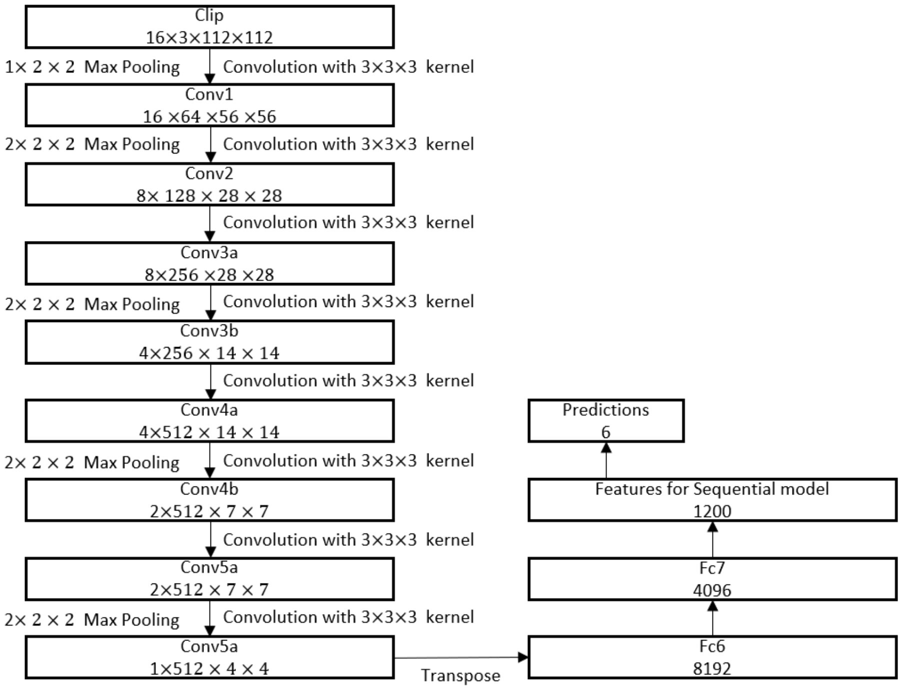

Fig. 1 The C3D Network Architecture with each box represting a tensor with the labeled size

2 Dataset Statistics

Video	Length (min)	Total Frames	Non-Phase (%)	Phase 1(%)	Phase 2(%)	Phase 3(%)	Phase 4(%)	Phase 5(%)	Transition Phase(%)
1	186	268083	5.59	2.83	50.49	32.08	1.36	6.56	1.10
2	224	322525	19.25	5.00	19.83	36.71	3.60	13.24	2.38
3	109	157850	11.21	12.71	25.98	29.91	3.21	12.54	3.43
4	107	153837	4.00	0.82	14.30	52.10	1.56	22.20	5.02
5	137	198146	9.08	3.32	26.98	38.37	0.58	9.71	11.96
6	536	426603	31.98	6.34	22.25	29.96	0.88	7.21	1.37
7	213	306329	9.53	3.49	33.88	31.64	2.19	12.57	6.70
8	154	221400	1.43	11.08	25.18	40.94	3.68	9.55	8.13
9	170	244958	11.53	5.46	15.05	48.00	1.68	12.06	6.21
10	189	271808	15.30	3.17	42.81	22.33	1.19	8.95	6.25
11	155	222804	6.47	4.98	23.94	44.58	0.46	14.92	4.64
12	216	310923	19.45	10.37	23.17	37.79	1.20	5.54	2.57
13	185	267171	1.19	8.18	44.38	28.23	5.55	7.10	5.38
14	123	177857	0.17	6.08	42.10	42.50	0.76	0.00	8.38
Average:	193	253592	10.44	5.99	29.31	36.80	1.99	10.15	5.25

Table 1 dataset Statistics
